# Diagnostic value of [^18^F]PSMA-1007 PET/CT based on PRIMARY score combined with mpMRI in clinically significant prostate cancer

**DOI:** 10.3389/fonc.2025.1589212

**Published:** 2025-06-18

**Authors:** Zhilong Ma, HaiTong Hao, Jian Chen, Tong Pan, Qian Zhao, YanMei Li

**Affiliations:** ^1^ Nuclear Medicine Department, General Hospital of Ningxia Medical University, Yinchuan, China; ^2^ College of Clinical Medicine, Ningxia Medical University, Yinchuan, China

**Keywords:** [18 F]F-PSMA-1007, positron emission tomography/computed tomography, multiparametric magnetic resonance imaging, prostate cancer, molecular probe

## Abstract

**Introduction:**

This study aimed to assess the diagnostic efficacy of the PRIMARY score, based on the ^18^F-labeled prostate-specific membrane antigen (PSMA-1007) positron emission tomography (PET)/computed tomography (CT) with multiparametric magnetic resonance imaging (mpMRI) PI-RADS, in detecting clinically significant prostate cancer (csPCa).

**Materials and Methods:**

In this retrospective cohort study, 137 patients with suspected prostate cancer (PCa) underwent [^18^F]PSMA-1007 PET/CT and mpMRI before transrectal ultrasound (TRUS)-guided needle biopsy was performed. Patients were categorized into csPCa and non-csPCa groups based on histopathological findings. The diagnostic performance of total prostate-specific antigen (TPSA), maximum standardized uptake value (SUVmax), the standardized Prostate Imaging Reporting and Data System (PI-RADS v2. 1) of mpMRI, and the PRIMARY score was evaluated using receiver operating characteristic (ROC) curves. The area under the curve (AUC), sensitivity, and specificity were calculated. Factors with a P-value <0.05 from the univariate analysis were included in a binary logistic regression model to develop a predictive model. Differences in the AUCs for TPSA, SUVmax, PI-RADS v2.1, the PRIMARY score, and the combined model were compared using MedCalc software. Statistical significance was set at P<0.05.

**Results:**

Among the 137 patients evaluated, 67.2% (92) were in csPCa and 32.8% (45) in the non-csPCa group (15 with low-grade PCa [GS 3 + 3] and 30 with benign prostatic hyperplasia or acute or chronic prostatitis). TPSA, SUVmax, PI-RADSv2.1, and the PRIMARY score significantly differed between the two groups (P<0.013). The AUCs for TPSA, SUVmax, PI-RADSv2.1, and PRIMARY score were 0.699, 0.898, 0.878, and 0.910, respectively, with corresponding diagnostic sensitivities of 53.3%, 87.0%, 90.2%, and 83.7%, and specificities of23.0%, 65. 1%, 42.6%, and 58.5%, respectively. The predictive ROC curve analysis of the model revealed an AUC of 0.968, with 91.3% sensitivity, and 84.6% specificity. MedCalc analysis showed that the AUC of the model was superior compared with that of SUVmax, PI-RADS v2.1 Score, and the PRIMARY score. The difference was statistically significant (Z= 2.273, 3.485, 2.761; P=0.023, 0.000, 0.005).

**Conclusions:**

The 5-grade PRIMARY score, derived from [^18^F]PSMA-1007 PET/CT in conjunction with the PI-RADSv2.1 score, offers enhanced discrimination of csPCa.

## 1 Introduction

Prostate cancer (PCa) is the second most widespread cancer and the fifth leading cause of cancer-related mortality in men, with approximately 1.5 million new cases worldwide by 2022 (1). According to clinical management and prognosis, PCa is classified into non-clinically significant (non-csPCa) and clinically significant (csPCa) categories (2). CsPCa was initially proposed by Epstein et al. and has since been widely adopted (3). It is pathologically defined using three criteria: an index tumor volume (ITV) >0.2 cm³, a Gleason score (GS) >7, or the presence of extracapsular extension (EPE) (4). These tumors are characterized by poor differentiation, high malignancy, and aggressive behavior, which necessitates prompt and active treatment (5). Conversely, non-csPCa refers to a tumor with GS <7, which is well-differentiated, has normal glandular structure and cell space, has low malignancy, weak invasiveness, and slow progression of the disease (6).

Consequently, active monitoring and regular follow-up are the main measures of non-csPCa; however, overdiagnosis and treatment may increase the burden on patients and reduce their quality of life (7). Therefore, the early discovery of csPCa helps to prolong overall survival and improve patient quality of life. Nevertheless, locating and differentiating between csPCa and non csPCa during prostate cancer screening remains challenging.

Previously, the total prostate-specific antigen (TPSA) was the most commonly used oncological marker for PCa screening. Nonetheless, TPSA levels can be identified in prostatitis, benign prostatic hyperplasia, and even following a digital rectal examination (DRE), which leads to a high rate of overdiagnosis and overtreatment of PCa (8). Transrectal ultrasound (TRUS)-guided needle biopsy is the primary method for diagnosing PCa. However, it has low test accuracy, leading to unnecessary prostate biopsies (which can induce sepsis) and high rates of overdiagnosis and overtreatment of non-csPCa (9).

Recently, multi-parametric magnetic resonance imaging (mp-MRI) has become the predominant non-invasive diagnostic technique for PCa. With the advancement of mp-MRI and the widespread implementation of the standardized Prostate Imaging Reporting and Data System (PI-RADSv2. 1), mp-MRI has shown high sensitivity and specificity in detecting csPCa. In the guidelines of the European Association of Urology and the National Institute for Health and Care Excellence in the UK, conducting mpMRI before prostate biopsy is recommended (10). Despite this, the positive predictive value of MRI remains relatively low, ranging from 34–68%, which leads to unnecessary biopsies. Furthermore, approximately 10–20% of csPCa remain undetectable using mp-MRI (11).

Prostate-Specific Membrane Antigen (PSMA) is a membrane glycoprotein, which is over-expressed in PCa cells. Research has shown that the expression of PSMA increases with increasing grade of PCa, rendering PSMA an ideal target for detecting PCa cells (12). PET/CT using radionuclide- labeled PSMA small-molecule inhibitors ([^68^Ga]Ga- PSMA - 11) has shown remarkable sensitivity and specificity in the diagnosis, staging, restaging, and prognosis assessment of prostate cancer (13, 14). The results of immunohistochemical studies also indicate a positive correlation between the expression level of PSMA and tumor grade. Reportedly, the imaging results of [^68^Ga]Ga-PSMA-11 PET/CT align with the degree of PSMA expression, and its maximum standardized uptake value (SUVmax) is significantly correlated with the International Society of Urological Pathology Grade Group (ISPP GG) of the primary tumor. These findings show that [^68^Ga]Ga- PSMA-11 PET/CT may be instrumental in predicting the occurrence of csPCa.

However, diagnosis of csPCa using PSMA PET/CT relies on a nuclear medicine physician’s experience and SUVmax values, which vary owing to different radionuclide markers and csPCa prevalence across institutions, limiting its prognostic accuracy (15). To address this, Emmett et al. introduced the PRIMARY score, a 5-grade system based on PSMA PET/CT, which improved diagnostic accuracy. At present, the PRIMARY score is a part of the standardized PCa Molecular Imaging assessment (16). Hence, the aim of this study was to explore whether combining the PRIMARY score based on [^18^F]PSMA-1007 PET/CT with mp-MRI enhances csPCa diagnostic efficacy.

## 2 Materials and methods

### 2.1 Participants

**Figure 1:**
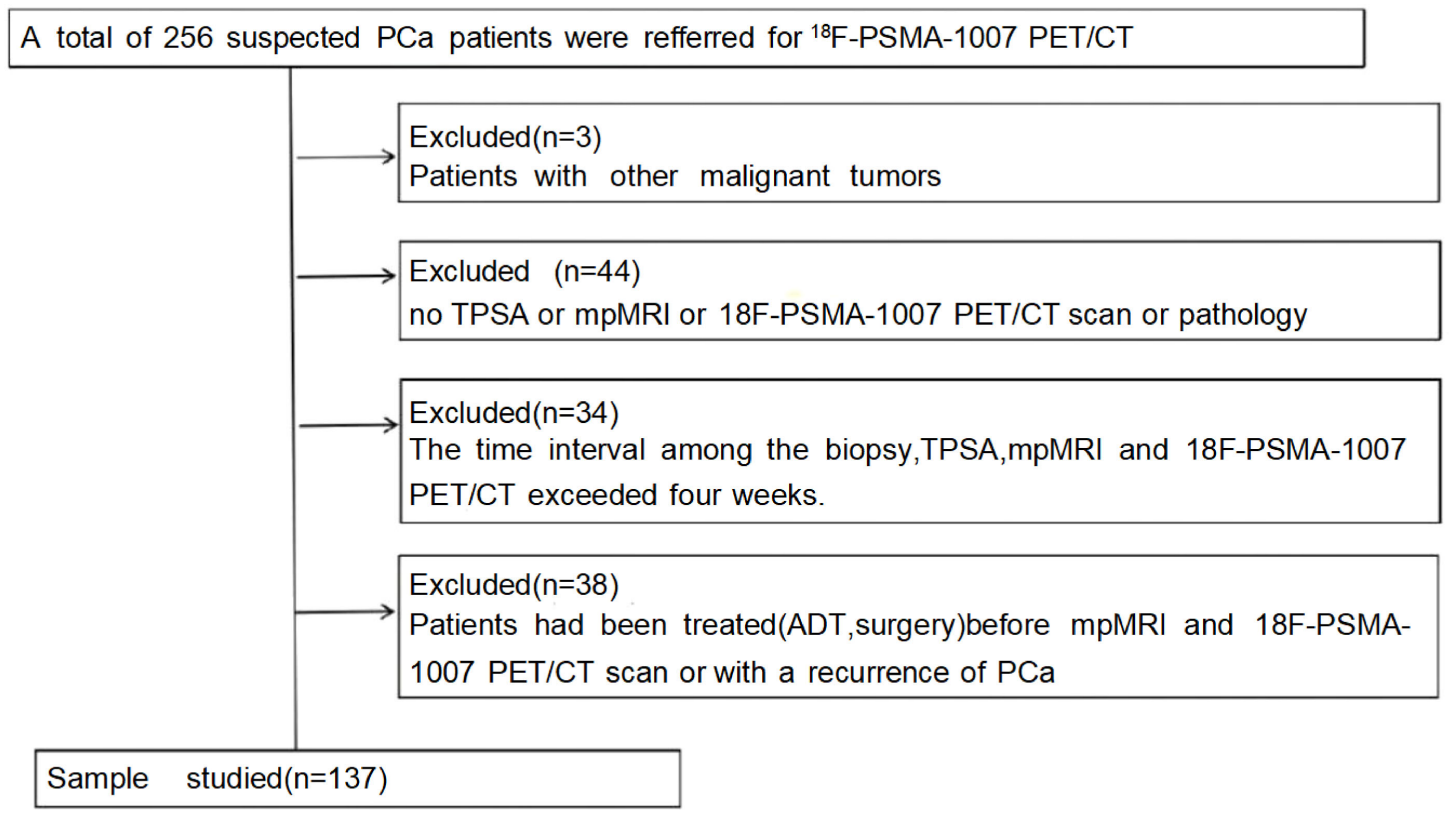
Flow chart of patient selection.

In this study, data from 256 patients who underwent [^18^F]PSMA-1007 PET/CT and mpMRI from January 1, 2020, to September 30, 2023, were retrospectively analyzed. Only those with complete data and who underwent TRUS or radical prostatectomy for pathological results were included, totaling 137 patients (**Figure 1**). Individuals meeting any of the following criteria were excluded from the study: 1) patients diagnosed with other malignant neoplasms. 2) without TPSA, mpMRI, or ^18^F-PSMA-1007 PET/CT scan or pathology. 3) >1 month between prostate biopsy, TPSA, mpMRI, and 18F-PSMA-1007 PET/CT scan. 4) those who had undergone treatment with first- or second-generation antiandrogen therapy, surgical intervention such as prostatectomy, or systemic therapy before imaging. The Hospital Ethics Committee approved the study (Approval No. 2020-083, 2020-876, dated January 16, 2020), adhering to the Declaration of Helsinki, with all participants providing informed consent.

### 2.2 Examination methods

#### 2.2.1 [^18^F]PSMA-1007 PET/CT scanning method

All patient examinations were conducted following the PCa PSMA PET/CT Imaging Guidelines, as outlined in the EANM Guidelines and SNMMI Procedure Standard 2.0. The [18F]PSMA-1007 PET/CT examinations were administered by a nuclear medicine technician with extensive experience and licensure for operating large-scale equipment. A GE Discovery VCT PET-CT scanner was used. The scanner was equipped with a 64-row CT, which met the necessary quality control standards. The ^18^F is produced by Sumitomo Japan’s cyclotron (HM-10), and the PSMA-1007 precursor was supplied by ABX Advanced Biochemical Compounds GmbH in Germany. The synthesis of [^18^F]PSMA-1007 was performed using the PET-IFB-X5 system, provided by Shaanxi Zhengze Biotechnology Co., Ltd. The compound’s purity was verified using high-performance liquid chromatography. The compound achieved a purity level of ≥95%. The administered dose of [^18^F]PSMA-1007 was 4.0 MBq/kg. A comprehensive whole-body scan was conducted approximately 60–90 min post-injection, followed by a spiral CT scan extending from the cranial roof to the mid-femur. The scanning parameters were set to a tube voltage of 140 kV, a tube current of 150 mA, a layer thickness of 3.75 mm, a pitch of 0.875, and a matrix size of 512×512. PET scanning was performed in a three-dimensional mode, with a scanning matrix of 128×128. The acquisition time for each bed position was 2.5 min, and 7–9 bed positions were acquired. CT data were employed for attenuation correction in the PET images, which were subsequently reconstructed and fused.

#### 2.2.2 mpMRI scanning method

The mpMRI examination was performed using a GE SIGNA™ Architect 3.0T MRI scanner and a 32-channel phased-array surface coil (GE). The scanning position was supine, with the center of the coil aligned 5 cm above the pubic symphysis. The scanning range was from the pubic symphysis to the bifurcation of the bilateral iliac blood vessels. Meanwhile, the scanning sequences included T2- weighted imaging (T2WI), T1-weighted imaging (T1WI), diffusion-weighted imaging (DWI), dynamic contrast-enhanced magnetic resonance imaging (DCE-MRI), and magnetic resonance image compilation (MAGiC) sequence. The DCE-MRI was used to scan 64-time phases (in total, 5 min and 12 s). In the second time phase, a bolus of the contrast agent gadodiamide (Omniscan, GE Healthcare AS) was injected into the elbow vein at a flow rate of 2.5 mL/s at a dose of 0.5 mmol/kg, and subsequently, 20 mL of normal saline was used for flushing.

### 2.3 Image analysis

#### 2.3.1 [^18^F]PSMA-1007 PET/CT image analysis

**Figure 2:**
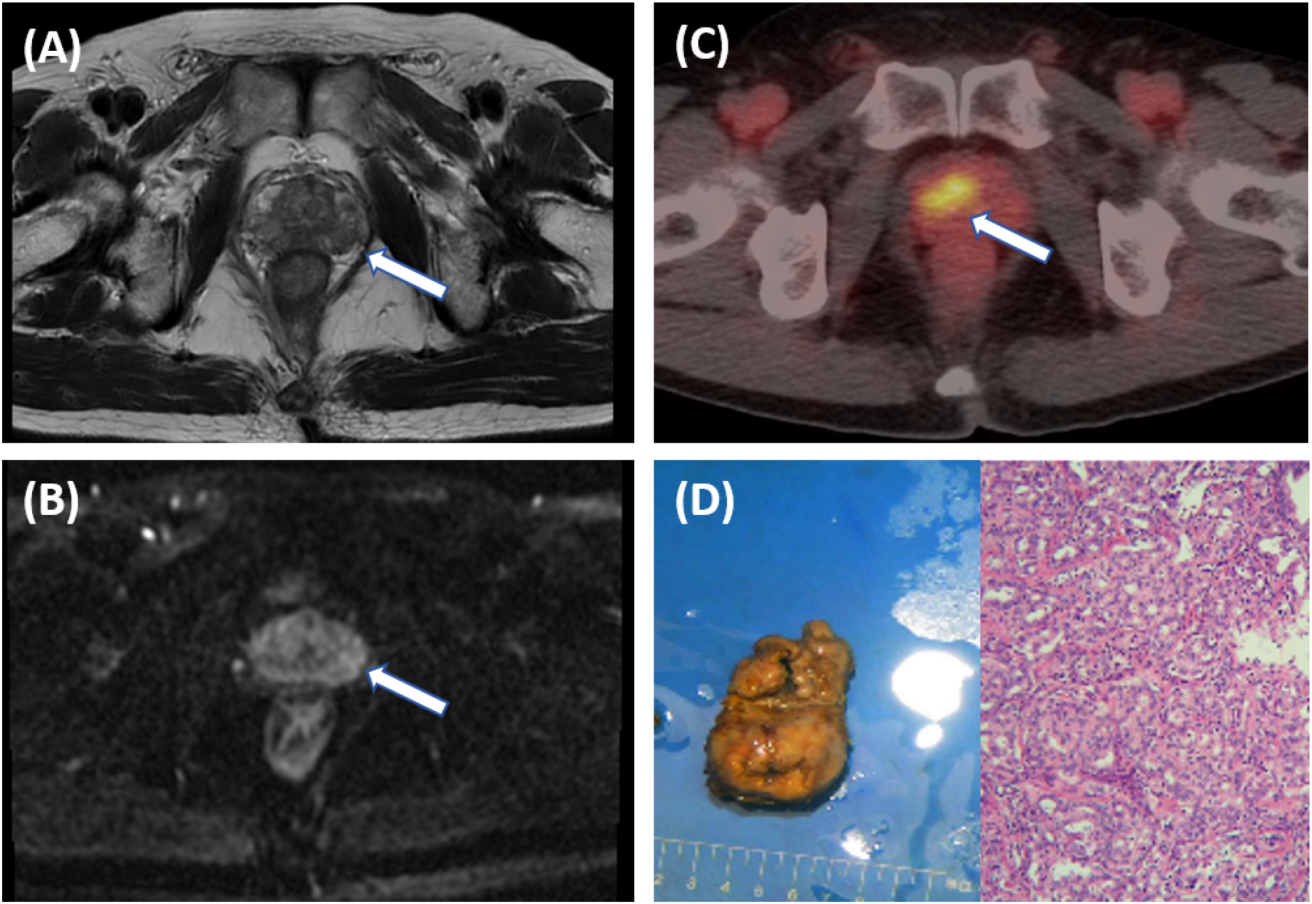
A 66-year-old male patient with an elevated PSA. Pelvic MRI shows multiple patchy T2 hypointense shadows in the bilateral peripheral zones of the prostate [**(A)**, arrow], and slightly high signal on DWI [**(B)**, arrow], with a PI-RADS score of 3, suggesting prostatitis. Further [18F]PSMA- 1007 PET/CT shows significantly increased PSMA expression on the right side of the middle region of the prostate [**(C)**, arrow], with SUVmax of 13.4, suggesting prostate cancer. The postoperative pathological results indicate prostate adenocarcinoma with a Gleason score of 3 + 5 = 8 **(D)**.

**Figure 3:**
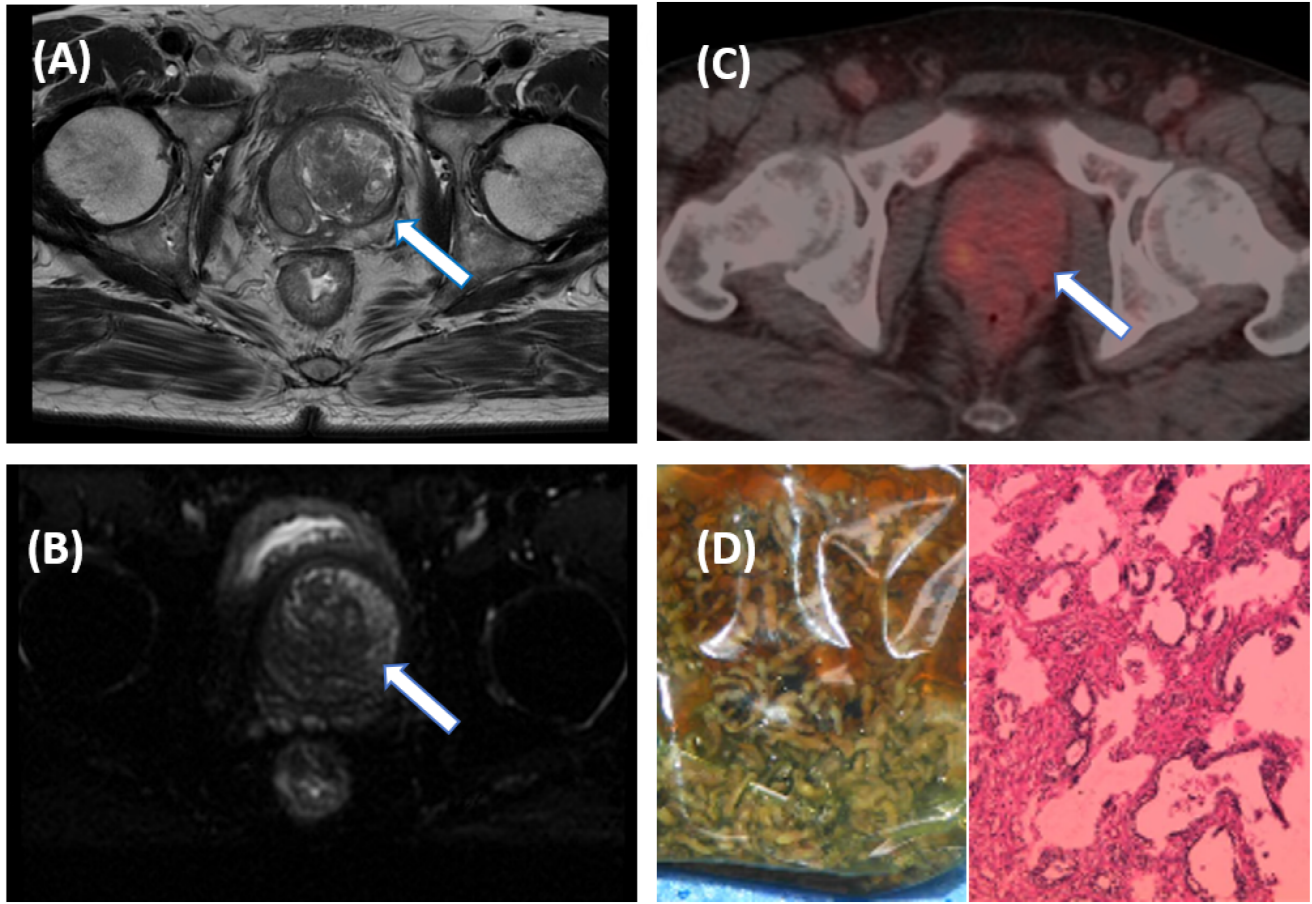
A 55-year-old male patient with an elevated PSA. Pelvic MRI shows multiple patchy T2WI hypointense lesions on the left side of the central zone of the prostate [**(A)**, arrow], and slightly high signal on DWI [**(B)**, arrow], suggestive of prostate cancer with a PI-RADS score of 5. Further [^18^F]PSMA-1007 PET/CT shows mildly increased PSMA expression in the central region of the prostate [**(C)**, arrow], with SUVmax of 4.2, suggesting prostate hyperplasia. The pathological results after electron microscopy surgery indicated prostate hyperplasia **(D)**.

**Figure 4:**
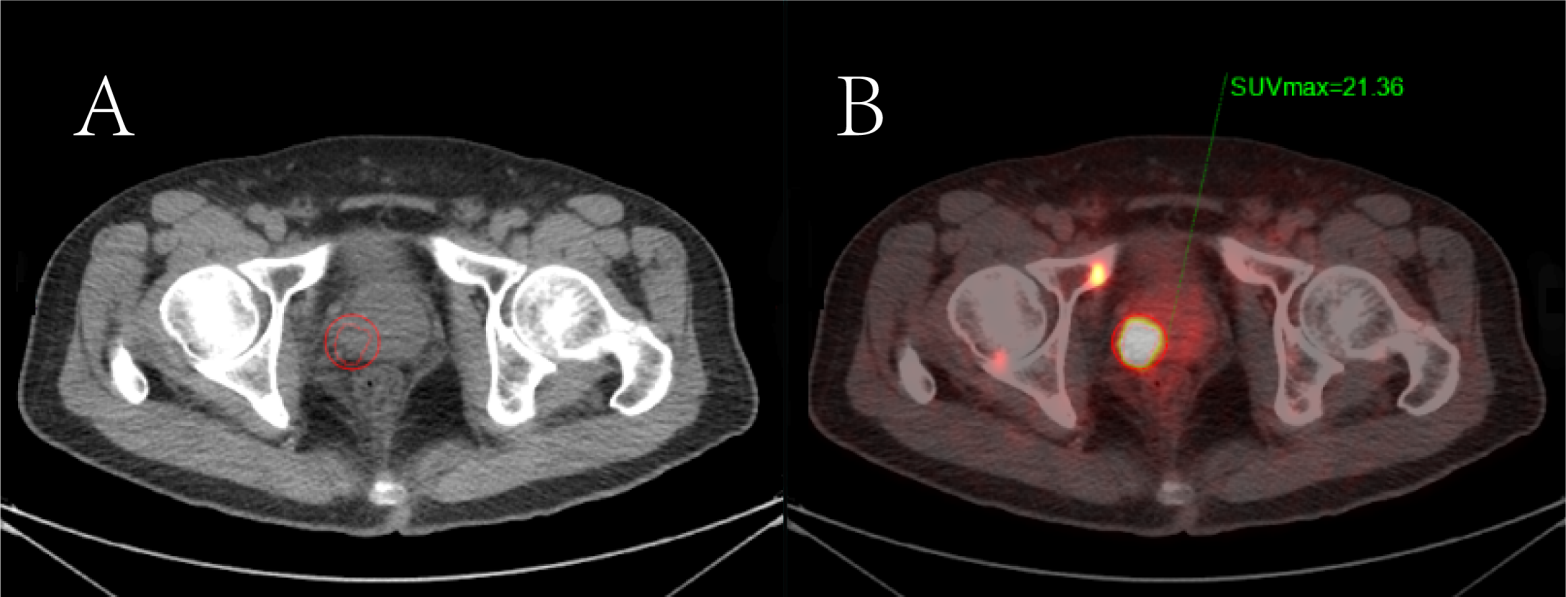
The semi-quantitative parameters of the primary prostate tumor measured by the 3D delineation method in [^18^F]PSMA-1007 PET/CT imaging. **(A)** Primary prostate cancer is shown on CT imaging; **(B)** The region of interest of the positive prostate lesion was obtained in the axial fusion image (the smaller red circle surrounding the lesion). The maximum standardized uptake value (SUVmax) of the lesion was obtained as 21.36 by the threshold method.

A double-blind methodology was used for the evaluation of the [^18^F]PSMA-1007 PET/CT scans, conducted by two nuclear medicine physicians (both with >10 years of experience) who were blinded to histopathological data and other imaging. The focus of the visual analysis was on the lesions demonstrating higher local uptake within the prostate compared with the adjacent prostatic tissue. (**Figures 2**, **3**) A circular region of interest was delineated at the axial level, and positive lesions within the prostate were identified using a fixed threshold method at 40% of the SUVmax (the acquisition method of SUVmax is shown in **Figure 4**).The PRIMARY score was assessed following the criteria established in the previous PRIMARY score study (17), which are as follows: score1 indicates no dominant intraprostatic pattern on PSMA with low-grade activity; score 2 denotes diffuse transition zone activity or symmetrical central zone activity that does not reach the prostate margin on CT; score 3 represents focal transition zone activity that is visually twice the background transition zone activity; score 4 corresponds to focal peripheral zone activity (with no minimum intensity requirement); and score 5 is characterized by a SUVmax >12. A PRIMARY score of 1–2 was classified as negative, whereas a score of 3–5 indicated a positive. Any discrepancies were resolved through consensus with a third nuclear medicine physician (with >10 years of experience).

#### 2.3.2 mpMRI image analysis

All MRI scans were processed using the Advantage Workstation 4.6 (GE Healthcare) and were routinely interpreted by specialized genitourinary radiologists (with 10 years of experience) based on PI-RADS v2.1. A PI-RADS score of 1–2 was classified as negative, while a score of 3–5 was classified as positive. Before analyzing the images, neither of the two doctors obtained the pathological data.

### 2.4 Pathological grading and gold standard

In patients who underwent radical surgery after the examination, the postoperative pathology served as the gold standard. Meanwhile, the pathology offline-needle aspiration biopsy was regarded as the gold standard for those who did not undergo radical surgery. The pathological grading of PCa was based on the GS system. Lesions with a GS≥7 (3 + 4) or an ITV>0.2 cm³, or the presence of EPE were defined as csPCa.

### 2.5 Statistical analysis

SPSS 26.0 (IBM Corp., Armonk, NY, USA) was used for the statistical analysis. The clinical and imaging data were descriptively analyzed. Normally distributed data are presented as mean ± standard deviation and compared using the independent sample t-test. The median (interquartile range) was used for non-normally distributed data. The receiver operating characteristic (ROC) curve was plotted to evaluate the diagnostic efficacy of TPSA, SUVmax, mpMRI PI-RADS v2.1, and the PRIMARY score for csPCa. The area under the curve (AUC), Youden’s index, sensitivity, and specificity were calculated. Univariate analysis was used to identify factors with P < 0.05 for inclusion in a binary logistic regression to create a predictive model. MedCalc was used to compare differences in TPSA, SUVmax, PI-RADSv2.1, PRIMARY score, and the predictive model’s AUC. Kappa statistic was employed to evaluate the consistency of the PRIMARY score. A kappa value ≥0.75 indicates good consistency, 0.4 ≤ kappa <0.75 suggests medium consistency, and kappa <0.4 suggests poor consistency. Statistical significance was set at P < 0.05.

## 3 Results

### 3.1 Clinical characteristics and general data

**Table 1** shows the general data of patients. Overall, 137 patients with a median age of 68 (range, 53–87) years were included. Among them, 67.2% (92/137) had csPCa, and 32.8% (45/137)) did not. The median TPSA was 22.6 (3.4–100) ng/mL, and the proportion of PI-RADSv2. 1 ≤ 2 and ≥3 scores were 13.9% (19/137) and 86. 1% (118/137), respectively. The proportion of the PRIMARY score from 1 through to 5 was 11.7% (16/137), 7.3% (10/137), 21.9% (30/137), 7.3% (10/137), and 51.8% (71/137), respectively.

**Table 1:** General data of the participants.

Characteristic	Numerical value
Age (years)	68 (53–87)
TPSA (ng/mL)	22.6 (3.4–100)
SUVmax	12.5(2.6-64.7)
PI-RADS Score	
1	5 (3.6%)
2	14 (10.2%)
3	18 (13.1%)
4	35 (25.5%)
5	65 (47.4%)
PRIMARY score	
1	16 (11.7%)
2	10 (7.3%)
3	30 (21.9%)
4	10 (7.3%)
5	71 (51.8%)
GS Score (%)	
No cancer	30 (21.9%)
6	15 (10.9%)
7	27 (19.7%)
8	26 (19.0%)
9	26 (19.0%)
10	13 (9.5%)

### 3.2 Comparison of patient data between csPCa and non-csPCa

The enrolled patients were categorized into two groups: csPCa and non-csPCa. The baseline clinical characteristics and imaging features were compared between the csPCa and non-csPCa groups using the independent sample t-test. The analysis revealed that the TPSA, SUVmax, PI-RADSv2.1, and PRIMARY score were significantly elevated in the csPCa group compared with those in the non-csPCa group, with t-values of 4.513, 7.078, 9.543, and 12.032, respectively, and a P-value <0.013, indicating statistical significance. Age did not differ significantly between the two groups (t = 3.915, P = 0.582) as presented in **Table 2**.

**Table 2:** Comparison of Age,TPSA, SUVmax, PI-RADS and PRIMARY Score between csPCa and non-csPCa.

Characteristic	csPCa (n=92)	ncsPCa (n=45)	t value	P value
Age	69.2±7.6	63.9± 7.7	3.915	0.582
TPSA (ng/ml)	40.0±31.3	18.5±8.6	4.513	0.000
SUVmax	17.3±7.5	7.6±7.3	7.078	0.001
PI-RADS (1~5)	4.5±0.9	3.0±1.1	9.543	0.013
PRIMARY Score (1~5)	4.5±0.9	2.3±1.1	12.032	0.000

### 3.3 ROC analysis of TPSA, SUVmax, PI-RADS v2.1, and PRIMARY score in the diagnosis of csPCa

**Figure 5:**
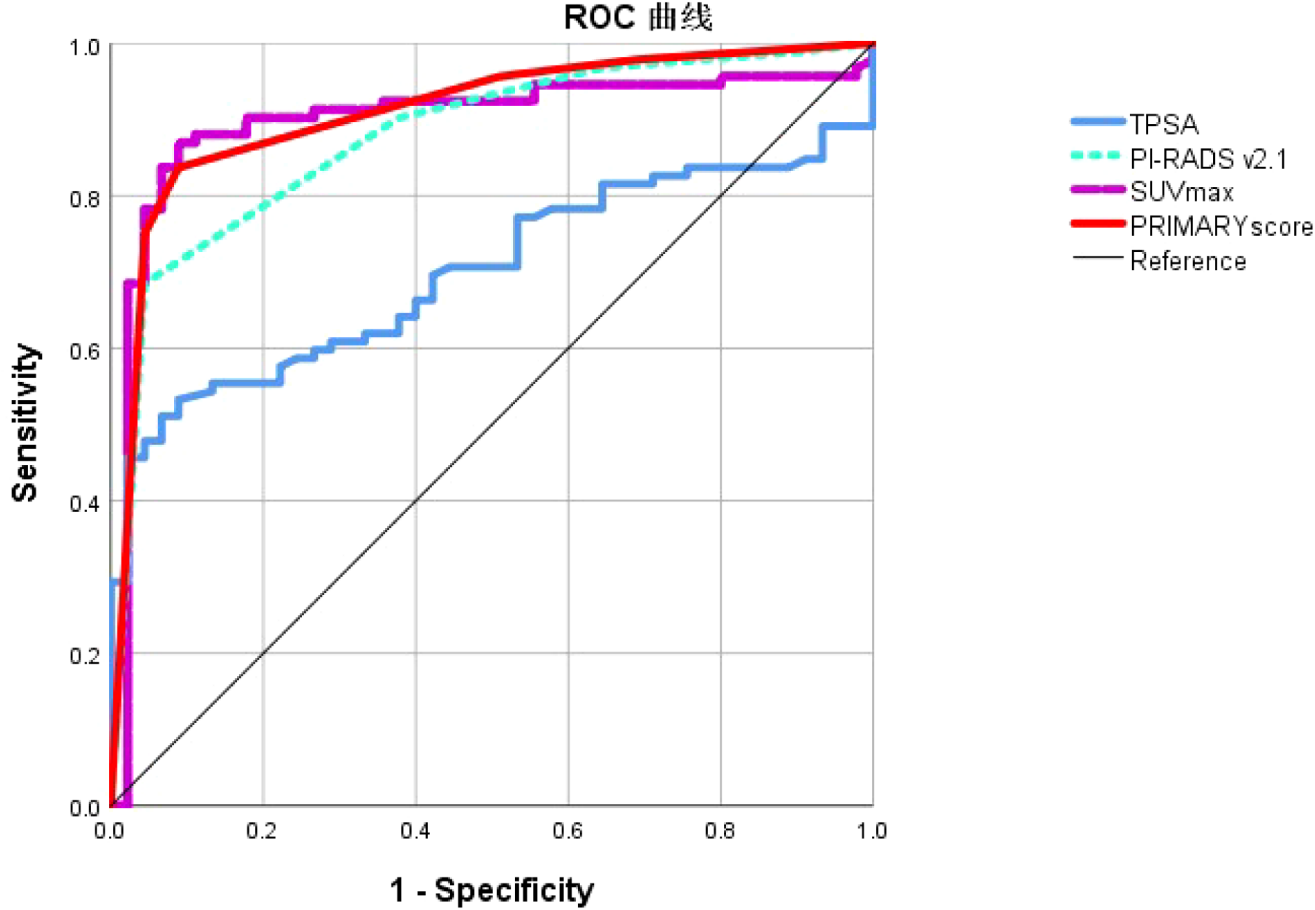
Receiver operating characteristic curve analysis of TPSA, SUVmax, PI-RADS v2.1 and PRIMARY score in the diagnosis of csPCa.

ROC curve analysis was employed to evaluate the diagnostic efficacy of TPSA, SUVmax, PIRADS, and PRIMARY scores for csPCa. The results showed that the AUC for TPSA, SUVmax, PIRADS, and PRIMARY scores in diagnosing csPCa were 0.699, 0.898, 0.878, and 0.910, respectively. The cut-off values were 29.9 ng/mL for TPSA, 10.02 for SUVmax, 4.5 for PI-RADSv2.1, and 3.5 for PRIMARY score, with corresponding diagnostic sensitivities of 53.3%, 87.0%, 90.2%, and 83.7%, and specificities of 23.0%, 65. 1%, 42.6%, and 58.5%, respectively (**Figure 5**, **Table 3**).

**Table 3:** ROC Results of TPSA, SUVmax, PI-RADS and PRIMARY Score for Diagnosing csPCa.

Method	Sensitivity	Specific ity	AUC	95% CI	p- value
TPSA(ng/ml)	53.3%	23.0%	0.699	0.614~0.784	0.000
SUVmax	87.0%	65.1%	0.898	0.836~0.961	0.000
PI-RADS(1~5)	90.2%	42.6%	0.878	0.818~0.938	0.000
PRIMARY Score(1~5)	83.7%	58.5%	0.910	0.857~0.963	0.000
Model	91.3%	84.6%	0.968	0.943~0.996	0.000

### 3.4 Construction and efficacy analysis of the csPCa combined diagnostic model

**Figure 6:**
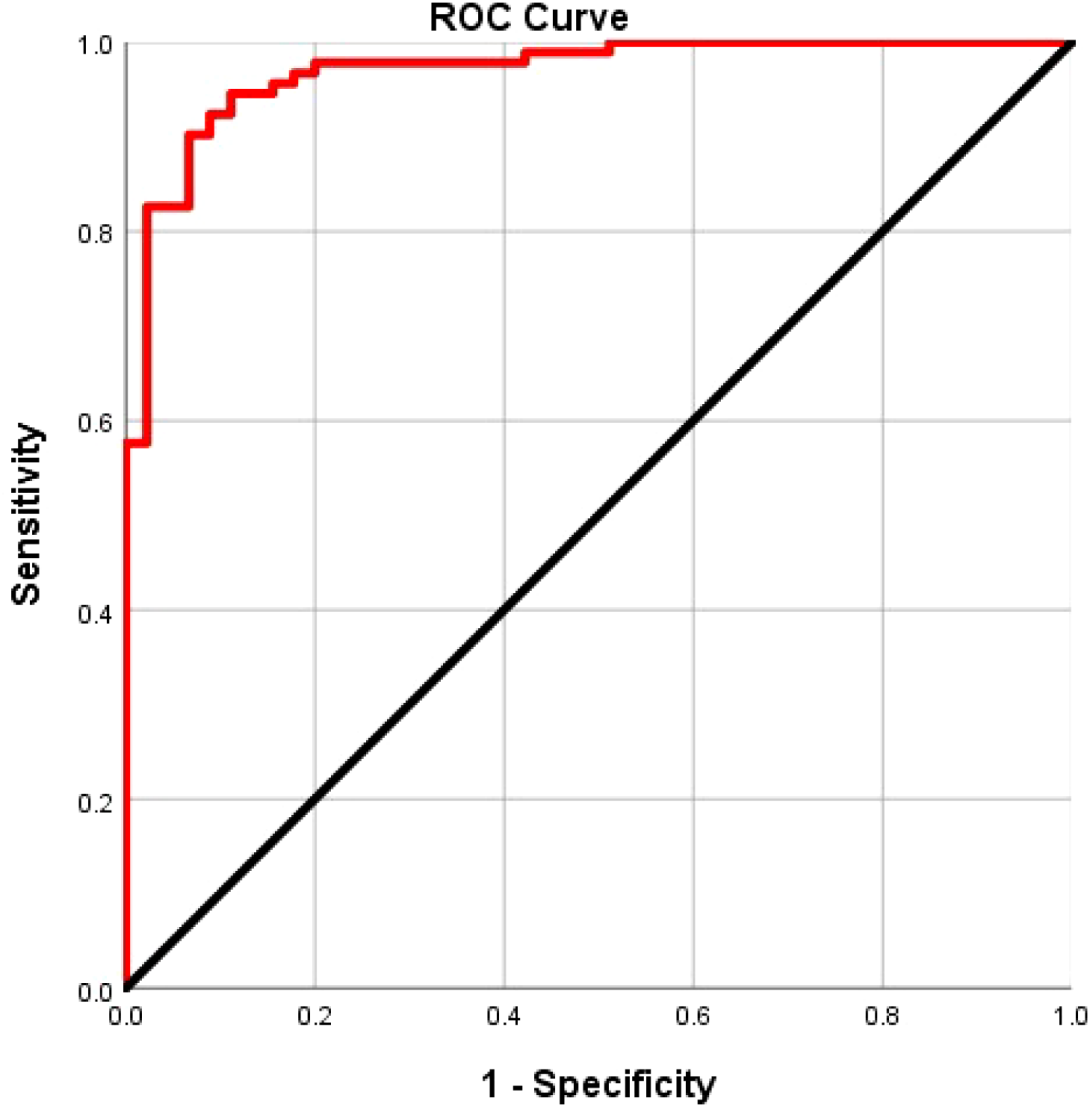
Receiver operating characteristic curve analysis of combined model for csPCa.

TPSA was excluded from the diagnostic model for csPCa because its AUC was <0.7. Instead, SUVmax, PI-RADS v2.1 score, and PRIMARY scores were used. The model’s ROC curve analysis revealed an AUC of 0.968, with 91.3% sensitivity, 84.6% specificity, and a Youden’s index of 0.835, outperforming the individual metrics (**Figure 6**). MedCalc findings were used to confirm the superior AUC of the model compared with that of SUVmax, PI-RADS v2.1 score, and PRIMARY score, with statistical significance (Z = 2.001, 2.167, 3.877; P = 0.048, 0.030, 0.000).

### 3.5 Consistency analysis of the PRIMARY scoring system among different raters

The Kappa test was used to evaluate the consistency of PRIMARY score among different raters. The results showed that there was a strong level of consistency between the results from the two raters, as evidenced by a kappa coefficient of 0.868.

## 4 Discussion

The csPCa is an aggressive malignancy with a prognosis that is generally not favorable, requiring early diagnosis and active treatment (18–20). Hence, predicting it early and accurately is of great significance for the treatment decision and prognosis of patients (12, 21). Findings from numerous studies indicate that PSMA PET/CT has impacted PCa diagnosis and treatment. High expression level of PSMA on PSMA PET/CT is positively correlated with GS, suggesting its predictive value for csPCa (22). In this study, the effectiveness of the PRIMARY score based on [^18^F]PSMA-1007 PET/CT, combined with mp-MRI for diagnosing csPCa, was assessed.

TPSA is not suitable for the diagnosis of csPCa. It was found in a study of415,000 individuals over 10 years that TPSA screening did not reduce PCa mortality but increased low-risk cancer detection, which often does not progress to csPCa (23). Our findings showed higher TPSA levels in csPCa cases, but with an AUC under 0.7, aligning with previous studies.

While mpMRI is preferred for csPCa detection, over 15% of cases are still missed using this approach (24). In PI-RADS 3 cases, significant uncertainty exists because csPCa is often below 20%, and over 50% of biopsied lesions are non-csPCa, indicating that using mpMRI-based PI-RADS scores may not fully and accurately diagnose prostate lesions (25, 26). Our findings revealed that the sensitivity of mpMRI for csPCa diagnosis was 68.5% with a specificity of 95.6%, possibly owing to benign lesions like prostatitis mimicking malignant signals. Furthermore, PI-RADS also relies on the expertise of the radiologist.

Superior efficacy was found using PSMA PET/CT in identifying PCa compared with using conventional imaging modalities. PSMA PET/CT shows greater potential for staging patients with csPCa (16, 27). Jiao et al. (28) in their study revealed that SUVmax of [^68^Ga]Ga-PSMA-11 PET/CT was used to differentiate csPCa from benign prostatic disease, revealing a cut-off value of 5.30, with a sensitivity of 85.85%, specificity of 86.21%. Recent studies show that [^68^Ga]Ga-PSMA PET/CT has similar accuracy to that of mpMRI for csPCa. Li et al. conducted a comparative analysis of the diagnostic capabilities of [^68^Ga]Ga-PSMA-617 PET/CT and mpMRI in a cohort of 67 patients with TPSA levels ranging from 4–20 ng/mL. The sensitivity and specificity of [^68^Ga]Ga-PSMA-617 PET/CT were 87.88% and 88.24%, respectively, while those of MRI were 84.85% and 52.94%, respectively, with AUC values of 0.881 and 0.689. The findings showed that [^68^Ga]Ga-PSMA-617 PET/CT has superior diagnostic efficacy compared with that of mpMRI (29).

However, the accuracy of PSMA PET/CT in diagnosing csPCa depends on the experience of nuclear medicine physicians and SUVmax values, which can differ because of various radionuclide markers and csPCa prevalence at different institutions, affecting its prognostic reliability.

Emmett et al. introduced a 5-point PRIMARY scoring system that integrates prostate region and PSMA expression patterns to enhance the capability of [^68^Ga]Ga-PSMA PET/CT in differentiating csPCa (17). Their findings revealed that the 5-grade PRIMARY score, when combined with the intraprostatic pattern and intensity, exhibited high diagnostic accuracy for csPCa.

The comparative effectiveness of SUVmax, PI-RADSv2.1, and PRIMARY score in distinguishing csPCa was evaluated in our study. The results indicated that the sensitivity of the SUVmax, PI- RADSv2.1, and PRIMARY scores were 87.0%, 90.2%, and 83.7%, with specificities of 65. 1%, 42.6%, and 58.5%, respectively. Respectively, their AUCs were 0.898, 0.878, and 0.910. There was no significant difference between the SUVmax, PI-RADSv2. 1, and PRIMARY scores (Z = 0.472,0.707, 0.877; P = 0.634, 0.381, 0.479). The PRIMARY score had a higher inter-rater agreement. Our findings contrast with those by Guo et al. (20), who demonstrated that the PRIMARY score outperformed PI-RADSv2.1 in detecting csPCa with an AUC of 0.873 compared with 0.786 for PI-RADSv2.1 (P < 0.001). This discrepancy may be attributed to differences in patient selection, because Guo et al. included a higher proportion of patients with PIRADS of 4 or 5, whereas more patients with PIRADS scores of 3 were included in our study. We included two patients with csPCa that did not express PSMA but had a PIRADS score of 5, which adversely affected the diagnostic accuracy of the SUVmax and the PRIMARY score. Furthermore, the incidence of PCa is associated with specific anatomical regions; however, using PSMA PET/CT has certain limitations in accurately localizing PCa anatomically. Variability in PET/CT instrumentation and different PSMA ligands may also contribute to uncertain anatomical localization with the PRIMARY score.

To enhance the differentiation of csPCa, we integrated SUVmax, PI-RADSv2.1, PRIMARY score, and a predictive model to improve the discriminatory capacity for csPCa. The findings indicated that the predictive model exhibited the highest predictive capability for csPCa, achieving an AUC of 0.968, with a sensitivity and specificity of 92.4% and 91. 1%, respectively. MedCalc showed that the predictive model’s AUC was significantly better than those of SUVmax, PI-RADS v2.1, and PRIMARY score (Z = 2.273, 3.485, 2.761; P = 0.023, 0.000, 0.005). The results showed that integrating the PRIMARY score with PI-RADS v2.1 enhanced sensitivity and improved diagnostic specificity. This improvement is primarily because of the increased detection rate of the MRI for csPCa lacking PSMA expression, while the PRIMARY score enhanced detection rates for PI-RADS 3 lesions.

A primary limitation of this study is its retrospective, single-center, small-sample design, which may have introduced bias owing to the limited number of patients with non-csPCa. Furthermore, the prediction model was not validated owing to the limited sample; thus, larger trials are necessary. Additionally, the use of prostate biopsy as the diagnostic gold standard may have resulted in missed diagnoses. Finally, we did not conduct a comparative analysis of the size and volume of prostate lesions between csPCa and non-csPCa, which may have influenced diagnostic outcomes; this will be addressed in subsequent studies.

The main strength of this study is the use of the latest PSMA PET/CT reporting system 5-PRIMARY score, combined with PI-RADS, to distinguish csPCa. Undeniably, combining both these approaches could enhance diagnostic accuracy even more. Meanwhile, it is important to consider that radiomics has emerged as a crucial area of research in recent years and represents a promising direction for future exploration. It facilitates the extraction of high-dimensional quantitative features from medical images, offering novel approaches for the quantitative analysis and precise diagnosis of tumor heterogeneity. In future research, we intend to develop and validate a model that integrates clinical factors, conventional parameters from [^18^F]PSMA PET/CT, and radiomics features to assess its potential utility in predicting csPCa prior to biopsy.

## 5 Conclusions

In conclusion, the 5-grade PRIMARY score, based on [^18^F]PSMA-1007 PET/CT combined with the PI-RADv2.1, can be used to more effectively distinguish csPCa, thereby reducing the likelihood of unnecessary biopsy procedures.

## Data Availability

The original contributions presented in the study are included in the article/supplementary material. Further inquiries can be directed to the corresponding authors.
